# Ethical Implications of Artificial Intelligence in Vaccine Equity: Protocol for Exploring Vaccine Distribution Planning and Scheduling in Pandemics in Low- and Middle-Income Countries

**DOI:** 10.2196/76634

**Published:** 2025-07-09

**Authors:** Ifeanyichukwu Akuma, Vina Vaswani, Elif Perihan Ekmekci

**Affiliations:** 1 Centre for Ethics Yenepoya Medical College Yenepoya (Deemed to be University) Dakshina City India; 2 Söğütözü Faculty of Medicine TOBB University of Economics and Technology Ankara Turkey

**Keywords:** algorithmic bias, digital inequity, digital inequities, ethical decision-making, vaccine logistics, health equity

## Abstract

**Background:**

The COVID-19 pandemic highlighted significant disparities in vaccine distribution, particularly in low- and middle-income countries (LMICs). Artificial intelligence (AI) has emerged as a potential tool to optimize vaccine distribution planning and scheduling. However, its ethical implications, including equity, transparency, bias, and accessibility, remain underexplored. Ensuring ethical AI implementation in vaccine distribution is crucial to addressing health equity challenges worldwide.

**Objective:**

This study aims to assess the ethical implications of AI-assisted vaccine distribution planning and scheduling in LMICs during pandemics. It seeks to evaluate AI’s role in ensuring equitable vaccine access, analyze ethical concerns associated with its deployment, and propose an ethical framework to guide AI-based vaccine distribution strategies.

**Methods:**

Our multiphase qualitative research approach will combine a systematic scoping review, a witness seminar with key stakeholders (health care professionals, AI developers, policymakers, and bioethicists), and a meta-synthesis of findings. The scoping review will follow PRISMA-ScR (Preferred Reporting Items for Systematic reviews and Meta-Analyses Extension for Scoping Reviews) guidelines, focusing on studies from 2019 to 2023. The witness seminar will provide firsthand insights into AI’s ethical impact on vaccine equity. Thematic content analysis and qualitative coding will be used for data interpretation, with findings integrated into a policy-driven ethical framework.

**Results:**

This study received institutional ethical approval in October 2023. Recruitment commenced in mid-August 2024 through email requests to prospective participants, and recruitment for the witness seminar (focus group discussion) is still ongoing, with 7 expert participants confirmed. Data collection is projected to conclude by August 2025. Preliminary literature analysis from the scoping review is ongoing, and qualitative data analysis from the witness seminar is scheduled for September 2025. The final results and proposed ethical framework are expected to be published in early 2026.

**Conclusions:**

By examining the ethical implications of AI in vaccine distribution, this research will provide actionable recommendations for policymakers, health care organizations, and AI developers. The findings will contribute to the discourse on responsible AI deployment in health care worldwide, ensuring transparency, fairness, and inclusivity in pandemic response strategies.

**International Registered Report Identifier (IRRID):**

DERR1-10.2196/76634

## Introduction

The COVID-19 pandemic, which emerged in 2019, exposed critical weaknesses in health care systems worldwide, particularly in the distribution and administration of vaccines. This crisis highlighted disparities in health care access, making vaccine equity a principle ensuring fair and just vaccine distribution a central concern [1]. Governments, international organizations, and health care authorities faced significant challenges in designing and implementing effective vaccine allocation strategies to ensure fair distribution across diverse populations [2,3]. Addressing these logistical challenges requires technology to play a pivotal role in supporting vaccine distribution efforts, and artificial intelligence (AI) is emerging as a promising tool for enhancing efficiency and equity. AI is applied in data analysis, pattern recognition, and predictive modelling [4,5]. AI could optimize vaccine distribution during pandemics by predicting outbreak hotspots, streamlining supply chains, and improving resource allocation. Its integration into vaccine planning and scheduling could improve both speed and fairness in distribution, particularly in resource-constrained settings.

However, vaccine equity is not solely a logistical issue; it also involves ethical and social considerations. Equitable vaccine access must account for socioeconomic status, geographic location, race, gender, and other intersectional identities [6]. During a pandemic, structural inequalities often result in marginalized populations experiencing greater barriers to vaccination [7]. Additionally, the increasing reliance on digital technology raises concerns about digital inequity, as disparities in internet access, digital literacy, and technological infrastructure can further limit vaccine access for disadvantaged communities [8,9]. Addressing these challenges requires a system that prioritizes vulnerable populations, responsibly leverages AI, and promotes inclusive vaccine distribution strategies.

The application of AI in vaccine distribution also introduces complex ethical dilemmas [10,11]. Questions regarding prioritization, transparency, accountability, privacy, and fairness arise in AI-driven decision-making. If not carefully designed, AI systems may reinforce biases, compromise trust, and shift critical health care decisions from human oversight to automated algorithms [4,12]. Digital platforms have been used for appointment scheduling, vaccine inventory tracking, cold-chain monitoring, and last-mile delivery coordination [4]. Geographic Information Systems (GISs) help map high-risk zones, while mobile apps assist in public outreach, reminders, and postvaccination surveillance [12]. In high-income settings, predictive analytics and cloud-based dashboards have enabled real-time decision-making. In low- and middle-income countries (LMICs), however, technological integration remains uneven due to infrastructure gaps, limited digital literacy, and resource constraints [8]. Nonetheless, several countries have piloted or adopted AI and machine learning tools for demand forecasting, route optimization, and prioritization models during the COVID-19 pandemic. These examples illustrate the promise and complexity of integrating advanced technologies into vaccine delivery systems. It is crucial to examine the ethical implications that emerge from such innovations, particularly in low-resource settings.

## Methods

### Study Design

This study will use a multiphase qualitative research design guided by an interpretivism approach [13], which emphasizes understanding the ethical implications of AI in vaccine distribution through the perspectives of key stakeholders. The study will integrate a systematic scoping review, a witness seminar [14,15], and a meta-synthesis [16], ensuring a comprehensive exploration of AI’s role in vaccine equity. Heterogeneous maximum variation sampling will be used to select diverse participants, including health care professionals, AI developers, policymakers, bioethicists, and vaccine recipients, to capture a broad spectrum of insights. The research aligns with Sustainable Development Goal 9 (SDG 9: Industry, Innovation, and Infrastructure) and SDG 10 (Reduced Inequalities) by examining how AI-driven vaccine distribution can enhance technological equity, while addressing disparities in health care worldwide. Through thematic content analysis and qualitative coding, the study aims to develop an ethical framework that promotes responsible and equitable AI implementation in pandemic response strategies. Although this study seeks to establish an ethical framework for AI-assisted vaccine distribution across LMICs, it is essential to note that it is intended to be exploratory and adaptable rather than universally prescriptive. The diversity among LMICs in terms of health care infrastructure, digital capacity, and sociopolitical contexts makes a one-size-fits-all model impractical. Instead, this study will produce a context-sensitive foundation (Indian context), informed by cross-sectoral perspectives, which can be refined and localized through future research. The witness seminar and metasynthesis are designed as initial steps in a phased approach, to generate core ethical considerations and actionable principles. Thus, although the scope is broad, the study explicitly recognizes its role as a starting point for iterative framework development, contributing foundational insights to the evolving discourse on ethical AI in public health logistics.

### Ethical Considerations

This study complies with ethical guidelines set by the Indian Council of Medical Research (ICMR) in 2017 and the World Medical Association Declaration of Helsinki (WMA DoH, 2013). Approval from the Scientific Review Board (SRB) of Yenepoya Medical College (Mangalore, Karnataka, India) was obtained on September 9, 2023, and ethical approval (YEC-1/2023/269) was granted by the college’s Institutional Ethics Committee (IEC) on May 10, 2023. All participants in the witness seminar will provide written informed consent after reviewing the participant information sheet (PIS), with the option to approve the edited transcript before publication. The study poses minimal-to-no risk, with no financial incentives, but offers academic contributions to public health ethics. Original recordings will remain confidential, while edited transcripts will be publicly available for research transparency.

### Study Plan

**Figure 1:**
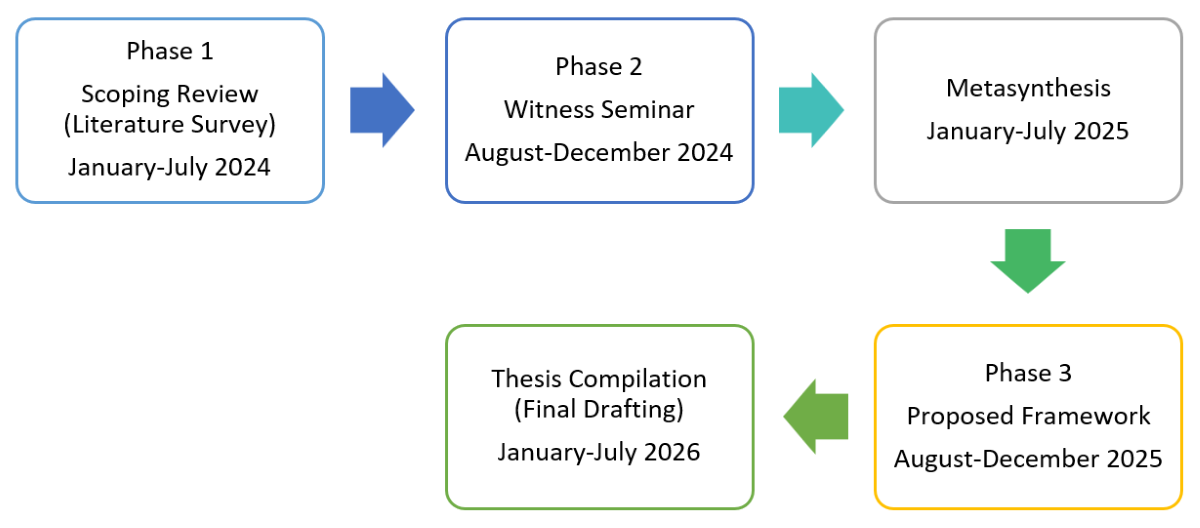
Study plan.

Figure 1 shows the study plan including the phases of this research.

#### Phase 1

The first phase of this study involves a systematic scoping review to analyze existing AI applications in vaccine equity, identifying key ethical considerations, challenges, and potential solutions. Following PRISMA-ScR (Preferred Reporting Items for Systematic reviews and Meta-Analyses Extension for Scoping Reviews) guidelines, the review will include full-text, open-access, peer-reviewed papers in English from PubMed, ScienceDirect, and the DOAJ, covering 2019-2023 (COVID-19 pandemic period). The study will focus on LMICs, examining how AI influences vaccine distribution planning and scheduling. Thematic analysis will use data from academic literature, government reports, and international health organizations. Heterogeneous maximum variation sampling will ensure diverse stakeholder perspectives, including policymakers and health care professionals. Papers will be screened based on predefined population, concept, and context (PCC) criteria, excluding gray literature and unpublished sources. The review will use string-based searches with AI-related keywords to ensure comprehensive coverage of AI-driven vaccine allocation and ethical dilemmas.

The inclusion criteria are as follows: studies (wide range of study designs or methods) involving stakeholders or systems related to vaccine distribution, in the English language, and with a time frame of 2019-2023. The exclusion criteria are as follows: papers that do not specify any LMIC-relevant context; do not discuss ethical issues, health equity, or decision-making implications; are published in languages other than English; or are not accessible in full text.

The search string includes the following terms: “AI-powered solutions” AND “Vaccine delivery” AND “Resource- constrained environments”; “AI ethics guidelines” AND “Vaccine logistics” AND “Equity in health care Ethics of AI applications” AND “Vaccine rollout” AND “Social justice”; “AI-driven vaccine allocation” AND “Ethical dilemmas” AND “Global health governance”; “Vaccine allocation ethics” AND “AI-based scheduling” AND “Health care disparities”; “AI-enabled scheduling” AND “Vaccine shortage” AND “Ethical decision-making”; “Artificial Intelligence applications” AND “Equitable vaccine access” AND “Low- and middle-income countries”; “Ethics of AI” AND “Pandemic response” AND “Vaccine scheduling”.

#### Handling of Duplicates and Data Management

**Figure 2:**
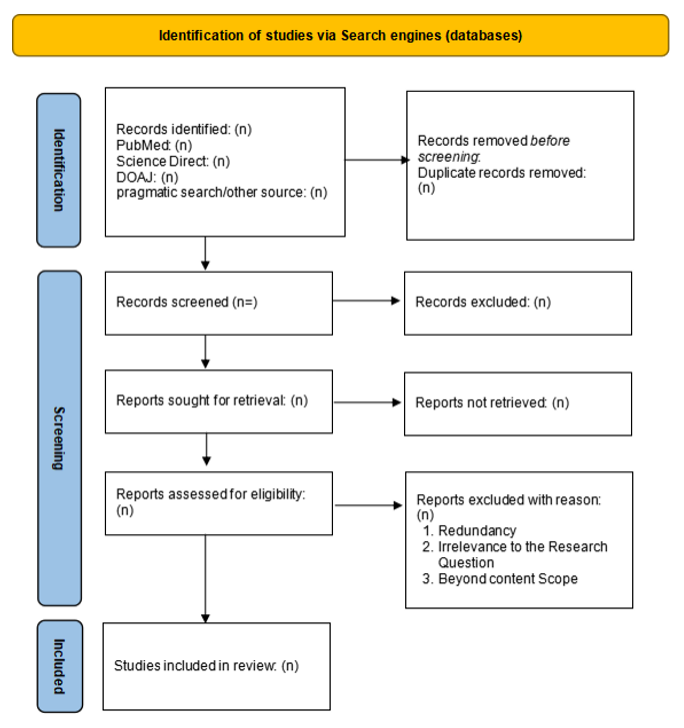
Screening process for paper selection based on PRISMA-ScR guidelines. PRISMA-ScR: Preferred Reporting Items for Systematic reviews and Meta-Analyses Extension for Scoping Reviews.

Search results will be imported into the Zotero reference manager to identify and remove duplicates. Extracted data will be charted using a developed data extraction sheet to record study objectives, AI applications, ethical concerns, and outcomes relevant to equity. The extracted data will then be analyzed thematically. A PRISMA-ScR flow diagram will be used to report the screening and selection process (Figure 2).

#### Phase 2

The second phase of this study will involve a witness seminar, bringing together key stakeholders, health care professionals, policymakers, AI developers, bioethicists, and vaccine recipients to share their firsthand experiences of AI’s role in vaccine distribution during the COVID-19 pandemic. As a historical event, the pandemic provides an opportunity to document ethical challenges related to privacy, data security, algorithmic bias, and the impact on marginalized communities in LMICs. The seminar will be transcribed and thematically analyzed to extract critical insights.

#### Phase 3

The third phase will involve developing an ethical framework for AI-supported vaccine distribution, using metasynthesis to integrate findings from the scoping review and witness seminar. Additionally, existing ethical frameworks and relevant case studies will be analyzed to shape a comprehensive model for responsible AI deployment in vaccine logistics. This multiphase qualitative and quantitative (qual/quant) study, conducted at Yenepoya (Deemed to be University) will span 18 months and rely on primary data collected from expert stakeholders.

### Sample Size

A total of 6-15 participants will be divided into 2 smaller groups, with each workshop comprising 5-7 participants at 2 time points. The participants will include prospective witness panelists selected for the seminar, ensuring alignment with the study’s research objectives and context. Participants will be chosen based on their expertise and direct relevance to AI-supported vaccine distribution, guaranteeing meaningful and insightful contributions. Although direct experience may be preferred, it is not mandatory. A smaller group size will allow for focused, in-depth discussions, facilitating a comprehensive exploration of diverse viewpoints, while maintaining high data quality. Additionally, resource constraints and feasibility considerations will make a smaller, well-curated panel more manageable within the available time frame and budget. The study will adopt a theoretical saturation approach, meaning data collection will continue until no new themes or ethical insights emerge during the seminar discussions. This will ensure that key patterns and perspectives are adequately captured despite the limited number of participants. Maximum variation sampling will be used to recruit stakeholders, emphasizing diversity in perspectives and fostering an environment of privacy and confidentiality, encouraging candid exchanges on AI’s ethical implications in vaccine equity. The emphasis on qualitative depth over breadth is consistent with witness seminar methodology, which prioritizes rich experiential narratives over statistical generalization. If thematic saturation is not achieved within the initial panel, a follow-up mini panel may be convened to address thematic gaps, ensuring rigor and completeness in capturing diverse ethical concerns.

### Recruitment

Maximum variation and purposive sampling will be used to recruit key stakeholders, including health care professionals, policymakers, AI developers, bioethicists, and vaccine recipients with relevant experience in AI-driven vaccine distribution, pandemic management, or health policy. Eligible participants must have at least 1 year of relevant experience, while those with less than a year’s or no relevant experience in, for example, vaccine distribution, digital health systems, or ethical policy discussions, will be excluded. Witness panelists may withdraw consent at any time, although their insights may still be used in the analysis after data are collected, based on the participants’ permission. Participants will be excluded if they decline to participate, fail to respond within 15 days, or face relocation or scheduling conflicts that prevent their involvement.

### Semistructured Interview Guide Development

A semistructured interview guide will be used as the primary research tool for the witness seminar (Tables 1 and 2). The guide will contain a list of open-ended questions designed to explore the ethical implications of AI in vaccine distribution. These questions are intended for clarity and flexibility, allowing for probing and in-depth discussions that align with the study’s objectives. There will be no fixed number of questions, as the inquiry process will emphasize knowledge and experience sharing among participants, including probes.

The interview schedule covers 5 key domains: AI in vaccine distribution, ethical implications of AI, vaccine distribution planning during pandemics, ethical considerations in vaccine scheduling, and equitable access to vaccines. Panelists will have the freedom to express their thoughts, emotions, and experiences to ensure a rich and nuanced discussion. To ensure validity, the interview schedule was reviewed by 3 experts, including 1 qualitative researcher and 2 bioethicists. Following validation, necessary revisions were made before dissemination via email. Additionally, the questionnaire underwent pretesting with 2 prospective panelists, who will not participate in the final seminar, to assess clarity, coherence, and language accuracy, ensuring the questions are easily understood and effectively elicit relevant responses.

**Table 1:** AI^a^-based distribution planning and scheduling systems’ effectiveness in ensuring equitable vaccine distribution in LMICs^b^.

Session	Description
Session 1: opening and introduction (5 minutes)	Brief overview of the seminar’s purpose, objectives, and format. Introduction of the panelists and their domain.
Session 2: COVID-19 vaccine access and vaccine equity (5-minute presentation to open up the discussion)	Each panelist presents their perspective on COVID-19 vaccine access and vaccine equity during the COVID-19 pandemic in LMICs (15 minutes).
Session 3: using AI support systems for vaccine scheduling and distribution (5-minute presentation to open up the discussion)	Each panelist presents their perspective on using AI support systems in vaccine distribution during the COVID-19 pandemic in LMICs (15 minutes).
Session 4: ethical implications of using AI support systems for vaccine scheduling and distribution and how it impacts equity (5-minute presentation to open up the discussion)	Each panelist presents their perspective on the ethical implications of AI support systems for vaccine scheduling and distribution and how using AI support impacted equity during the COVID-19 pandemic in LMICs (15 minutes).
Break (10 minutes)	Break to refresh
Session 5: health equity toward responsible AI implementation (5-minute presentation to open up the discussion)	Panelists share recommendations for responsible AI implementation in vaccine distribution to ensure health equity and ethical considerations (15 minutes).

^a^AI: artificial intelligence.

^b^LMIC: low- and middle-income country.

**Table 2:** Interactive discussion questions (based on the sessions) for the focus group interview.

Session	Questions
Session 2: COVID-19 vaccine access and vaccine equity	How can AI^a^ be leveraged to enhance vaccine equity and promote equitable vaccine distribution (eg, registration, appointment scheduling, and outreach)? How might integrating AI into vaccine scheduling and distribution enhance access and equity? How will AI-based systems be made to recognize and address specific vulnerabilities of groups or biases in the processes that affect equitable distribution? How do AI-driven approaches impact the decision-making process for prioritizing vaccine distribution? How do you think AI can contribute to promoting vaccine equity?
Session 3: using AI support systems for vaccine scheduling and distribution	What potential challenges or limitations exist in implementing AI-driven approaches to enhance health ethics worldwide in promoting vaccine equity?
Session 4: ethical implications of using AI support systems for vaccine scheduling and distribution and how it impacts equity	What potential ethical challenges do you foresee in implementing AI-driven approaches for vaccine equity (eg, individual privacy concerns, transparency, accountability, potential biases or inequalities, marginalization, cultural and social integration)? What steps should be taken to address these limitations or unintended consequences that may arise from AI-driven approaches in vaccine distribution?
Session 5: health equity toward responsible AI implementation	What are the perceptions and attitudes of individuals toward AI-driven approaches in promoting vaccine equity, and how do these perceptions influence their willingness to participate?

^a^AI: artificial intelligence.

#### Semistructured Interview Administration

The witness seminar will be conducted in a structured and interactive format to gather insights from expert panelists on the ethical implications of AI in vaccine equity during pandemics. Invitations will be sent to selected stakeholders, including health care professionals, policymakers, AI developers, bioethicists, and vaccine recipients, who have firsthand experience of AI-based vaccine distribution. The seminar will be held in hybrid mode, with the physical venue at Demo Room II, 3rd Floor, Centre for Ethics, Yenepoya (Deemed to be University), and a Zoom link for online participants. To ensure informed discussions, panelists will receive background reading materials before the event, with downloadable links provided during registration. A question schedule will guide discussions, prompting participants to share their experiences, ethical concerns, and recommendations regarding AI-driven vaccine allocation.

The entire session will be recorded with participant consent, and comprehensive notes will be taken to capture key insights, perspectives, and areas of agreement or disagreement. Following the seminar, the recordings will be transcribed, and a thematic analysis will be conducted to identify emerging patterns and ethical concerns. Panelists will get an opportunity to review and verify the accuracy of the findings before dissemination. The final report, including key discussion points and recommendations, will be shared with stakeholders, policymakers, and the broader research community through publications, presentations, or policy briefs. This process will ensure transparency, accuracy, and meaningful contributions to the ongoing discourse on ethical AI implementation in vaccine distribution.

### Data Analysis Plan

A holistic approach will be used to analyze the qualitative data, providing a comprehensive understanding of AI’s impact on vaccine equity, distribution, planning, and scheduling during pandemics. The witness seminar transcripts will undergo thematic content analysis, identifying key themes, patterns, and ethical concerns related to AI-driven vaccine allocation in LMICs. Thematic insights will be extracted through focused coding, while summative content analysis will be used to map responses against established ethical principles. The principal investigator (PI) will independently conduct the initial analysis, which will then be reviewed and compared with findings from the research guides (coinvestigators) to ensure accuracy and consistency.

Although the study is primarily qualitative, it incorporates a limited quantitative descriptive component in the form of (1) trend analysis from the scoping review (eg, publication frequency by year, geographical distribution of studies, use of AI methods) and (2) a code frequency during the witness seminar. These data will be analyzed descriptively to identify frequency patterns and dominant ethical themes. The quantitative findings will be used to triangulate and support the thematic insights from qualitative coding, aligning with a qual/quant-embedded design, where qualitative data are dominant and quantitative data play a supportive role.

The qualitative data collected through the witness seminar will be analyzed using a thematic analysis approach, specifically following the 6-phase framework by Braun and Clarke [17]: (1) familiarizing with data, (2) generating initial codes, (3) searching for themes, (4) reviewing themes, (5) defining and naming themes, and (6) producing the report. Transcripts will be coded both inductively and deductively, with predefined ethical concepts guiding initial coding, while allowing for the emergence of novel themes. To enhance intercoder reliability, 2 researchers will independently code a subset of the data. Discrepancies will be resolved through discussion, and a third reviewer will adjudicate unresolved differences. Cohen κ>0.70 will be considered acceptable for intercoder agreement. A codebook will be maintained throughout, and regular peer debriefing sessions will be held to ensure consistency.

Validation of findings will involve member checking, where participants will review the analyzed summaries to verify the accuracy of their perspectives; triangulation of findings from the scoping review and witness seminar to cross-validate emerging themes; and audit trails documenting decisions made during analysis, ensuring transparency and reproducibility. Biblioshiny and R (R Foundation for Statistical Computing) will be used strictly for the descriptive and bibliometric analysis of the scoping review phase (eg, tracking publication trends, co-occurrence of keywords, and country-wise contributions). These quantitative insights will support, but not replace, the primary qualitative thematic findings.

### Trustworthiness of the Study

Ensuring the trustworthiness of this study is critical to establishing the rigor, reliability, and validity of the findings. This will be achieved through credibility, transferability, dependability, and confirmability, supported by multiple verification techniques.

#### Credibility

The study will incorporate triangulation, member checking, and negative case analysis. Triangulation will be achieved through a multiphase approach, integrating data from the systematic scoping review, witness seminar, and metasynthesis to ensure a comprehensive understanding of AI’s ethical implications in vaccine equity. Member checking will allow witness seminar participants to review edited transcripts and verify the accuracy of their statements before finalizing the analysis. Additionally, negative case analysis will be conducted by identifying divergent perspectives on AI implementation in vaccine distribution, ensuring that all viewpoints are represented.

#### Transferability

The study will provide a reasonable description of the research context, including detailed participant experiences, ethical challenges, and AI’s impact on vaccine distribution in LMICs. Through the documentation of specific factors influencing AI-based vaccine allocation, the findings can be applied in similar settings worldwide.

#### Dependability

The study will ensure dependability through an audit trail, systematically documenting all research steps, from protocol development to data collection, analysis, and reporting. A structured data management approach will be followed, with the PI and research guides independently reviewing findings to ensure consistency and replicability.

#### Confirmability

The research process will be transparent and objective, minimizing potential researcher bias. The audit trail will maintain records of data collection methods, coding processes, and analysis decisions to allow external validation.

#### Reflexivity

A reflexive approach will be maintained throughout the study to acknowledge and address potential researcher biases. This will involve continuous self-evaluation and critical reflection on how the researchers’ backgrounds, perspectives, and assumptions may shape data interpretation.

## Results

This study received ethical approval in October 2023. As of June 2025, recruitment for the witness seminar is actively ongoing, with 7 panelists confirmed, including representatives from public health policy, bioethics, and AI development sectors. The scoping review phase is in progress, and literature analysis is yet to be finalized. Data collection for the witness seminar is projected to be completed by August 2025, with data analysis and synthesis planned for September-October 2025. Final findings, including the proposed ethical framework, are expected to be submitted for publication in early 2026.

## Discussion

### Summary

This study is expected to reveal that AI-based systems could offer logistical advantages in vaccine distribution, such as predictive modelling, scheduling, and supply chain management. However, these systems also raise significant ethical concerns. Anticipated themes include algorithmic bias, data privacy risks, marginalization due to digital inequities, and a lack of transparency in AI decision-making processes [18-21]. Although previous studies have examined AI applications in health care logistics and ethical issues in digital health, few have explicitly focused on the intersection of blockchain and vaccine distribution. This study fills that gap by triangulating evidence from the literature and lived experiences of diverse stakeholders. Moreover, it distinguishes itself by using a witness seminar method, a novel qualitative approach for capturing real-time policy-relevant ethical narratives.

### Strengths and Limitations

A significant strength of this study is its multimethod design, integrating a scoping review, a witness seminar, and a metasynthesis to enhance validity and triangulation. Additionally, including multiple stakeholder types ensures a holistic understanding of the ethical landscape. However, the study is limited by its small sample size and recruitment from India in the witness seminar, which, despite efforts to reach data saturation, may not fully capture the diversity across all LMIC contexts. Furthermore, ethical frameworks developed through qualitative synthesis may require further field validation in specific countries or communities to test relevance and applicability.

Based on this protocol, follow-up studies will focus on country-specific validation of the ethical framework and development of context-sensitive AI governance tools for public health use in LMICs. Engagement with policymakers, ethicists, and AI developers will be crucial to refine and scale ethical guidelines in future pandemics. The findings will be shared through peer-reviewed publications, academic conferences, and policy briefs targeted at public health authorities, AI developers, and health organizations worldwide. Stakeholder engagement sessions and webinars will also be conducted to translate the ethical framework into actionable guidance. All outputs will be made available via open access platforms to support transparency and uptake in LMIC contexts.

### Conclusion

This study will explore the ethical implications of AI in vaccine distribution, particularly in LMICs, where access disparities persist, integrating a systematic scoping review, a witness seminar, and a metasynthesis to provide insights into both potential benefits and ethical challenges of AI-driven vaccine allocation. The findings could inform policymakers and health care professionals, offering practical recommendations for responsible AI deployment in pandemic response efforts. Additionally, the study aims to develop an ethical framework to guide equitable and transparent AI implementation in vaccine distribution. Through stakeholder engagement and policy recommendations, this research will contribute to ethical AI governance in health care worldwide. The generalizability of findings to different LMICs may pose limitations. However, this study can serve as a model for new learnings, offering valuable insights that can be adapted and refined for similar research and policy development.

## Abbreviations

AI: artificial intelligence
LMIC: low- and middle-income country
PRISMA-ScR: Preferred Reporting Items for Systematic reviews and Meta-Analyses Extension for Scoping Reviews
PI: principal investigator
qual/quant: qualitative and quantitative
SDG: Sustainable Development Goal

## Data Availability

The data generated and analyzed during the interviews will be made publicly available upon reasonable request, but as of this protocol publication, no data are available.

## References

[1] Kontogiannis T (2021). A qualitative model of patterns of resilience and vulnerability in responding to a pandemic outbreak with system dynamics. Safety Sci.

[2] Parveen M (2020). Challenges faced by pandemic COVID 19 crisis: a case study in Saudi Arabia. Challenge.

[3] Hammami R, Salman S, Khouja M, Nouira I, Alaswad S (2023). Government strategies to secure the supply of medical products in pandemic times. Eur J Operat Res.

[4] Das AK, Bera B, Giri D (2021). AI and blockchain-based cloud-assisted secure vaccine distribution and tracking in IoMT-enabled COVID-19 environment. IEEE Internet Things Mag.

[5] Chowdhury D, Poddar S, Banarjee S, Pal R, Gani A, Ellis C, Arya RC, Gill SS, Uhlig S (2022). CovidXAI: explainable AI assisted web application for COVID-19 vaccine prioritization. Internet Technol Lett.

[6] Gómez-Ramírez O, Iyamu I, Ablona A, Watt S, Xu AXT, Chang H, Gilbert M (2021). On the imperative of thinking through the ethical, health equity, and social justice possibilities and limits of digital technologies in public health. Can J Public Health.

[7] Ahmed A, Lundahl M, Wadensjö E (2023). Ethnic discrimination during the COVID-19 pandemic. Migration and Integration in a Post-Pandemic World.

[8] Zheng Y, Walsham G (2021). Inequality of what? An intersectional approach to digital inequality under Covid-19. Inf Org.

[9] Imran A (2022). Why addressing digital inequality should be a priority. E J Info Sys Dev Countries.

[10] Grady C, Shah S, Miller F, Danis M, Nicolini M, Ochoa J, Taylor H, Wendler D, Rid A (2020). So much at stake: ethical tradeoffs in accelerating SARSCoV-2 vaccine development. Vaccine.

[11] Gur-Arie R, Jamrozik E, Kingori P (2021). No jab, no job? Ethical issues in mandatory COVID-19 vaccination of healthcare personnel. BMJ Glob Health.

[12] Khan JI, Khan J, Ali F, Ullah F, Bacha J, Lee S (2022). Artificial intelligence and internet of things (AI-IoT) technologies in response to COVID-19 pandemic: a systematic review. IEEE Access.

[13] Keane E, Thornberg R (2024). Grounded theory and constructivist grounded theory in educational research. The Routledge International Handbook of Constructivist Grounded Theory in Educational Research.

[14] Maas H, Düppe T, Weintraub ER (2020). The witness seminar: method, results, and implications. A Contemporary Historiography of Economics.

[15] Nicholls EJ (2020). The witness seminar: a research note. QuaL Res.

[16] Walsh D, Downe S (2005). Meta-synthesis method for qualitative research: a literature review. J Adv Nurs.

[17] Braun V, Clarke V (2006). Using thematic analysis in psychology. Qual Res Psychol.

[18] Asundi A, O'Leary C, Bhadelia N (2021). Global COVID-19 vaccine inequity: the scope, the impact, and the challenges. Cell Host Microbe.

[19] Buccieri K, Gaetz S (2013). Ethical vaccine distribution planning for pandemic influenza: prioritizing homeless and hard-to-reach populations. Public Health Ethics.

[20] Van De Pas R, Widdowson M, Ravinetto R, N Srinivas P, Ochoa TJ, Fofana TO, Van Damme W (2022). COVID-19 vaccine equity: a health systems and policy perspective. Expert Rev Vaccines.

[21] Yarlagadda H, Patel MA, Gupta V, Bansal T, Upadhyay S, Shaheen N, Jain R (2022). COVID-19 vaccine challenges in developing and developed countries. Cureus.

